# Tissue specific role of ABCA1 in lung cholesterol homeostasis under high-cholesterol diet

**DOI:** 10.3389/fnut.2025.1649407

**Published:** 2025-07-30

**Authors:** Jian Ma, Zhongwen Gong, Hong Lu, Han Yang, Shengquan Wang, Qian Zhu, Hongya Liu, Yongjia Li, Yuemei Zhang, Xuemei Lian

**Affiliations:** ^1^Center for Lipid Research & Chongqing Key Laboratory of Metabolism on Lipid and Glucose, Key Laboratory of Molecular Biology for Infectious Diseases (Ministry of Education), The Second Affiliated Hospital, Chongqing Medical University, Chongqing, China; ^2^Department of Nutrition and Food Hygiene, College of Public Health, Chongqing Medical University, Chongqing, China; ^3^Clinical Nutrition Department of Nantong University Affiliated Hospital, Nantong, China; ^4^Clinical Medical Research Center, Panzhihua Central Hospital, Panzhihua, China

**Keywords:** ABCA1, cholesterol homeostasis, lung metabolism, high-cholesterol diet, tissue specificity

## Abstract

**Background:**

ATP-binding cassette subfamily A1 (ABCA1) and sterol 27-hydroxylase (CYP27A1) are essential regulators of cholesterol metabolism. However, their tissue-specific roles, particularly in the lung, under high-cholesterol diet (HCD) conditions remain unclear.

**Objective:**

Using the liver as a reference, this study aimed to investigate the tissue-specific regulation of ABCA1 in the lung under HCD or CYP27A1 knockout (KO) conditions, and to explore its potential regulatory mechanism.

**Methods:**

CYP27A1 KO and wild-type (WT) mice on a C57BL/6J background were fed either a normal diet (ND) or HCD for 12 weeks. Transcriptome sequencing (RNA-seq) was conducted on lung tissue samples.

**Results:**

HCD feeding in WT mice caused significant hepatic lipid accumulation, while no notable lipid deposition was observed in lung tissue. ABCA1 and CYP27A1 expression were downregulated in the liver but upregulated in the lung. In *CYP27A1*^(−/−)^ mice, hepatic lipid accumulation was more severe with further suppression of ABCA1, whereas ABCA1 expression in the lung remained elevated. Transcriptome analysis revealed that upregulated genes in lung tissue were significantly enriched in the inflammation-related nuclear factor kappa-B (NF-κB) signaling pathway. Furthermore, experiments confirmed that the expression of NF-κB pathway was consistent with the upregulation of ABCA1.

**Conclusion:**

ABCA1 exhibits marked tissue specificity under HCD feeding or CYP27A1 KO conditions. In the liver, ABCA1 downregulation may exacerbate cholesterol metabolic imbalance, while its upregulation in the lung may play an important role in maintaining cholesterol homeostasis. Moreover, the increase in pulmonary ABCA1 expression in CYP27A1 KO mice may be associated with activation of the NF-κB signaling pathway.

## 1 Introduction

Cholesterol is an important component of mammalian cell membranes and has a significant impact on the structure and function of cell membranes (1). In addition, cholesterol is also a precursor substance for the synthesis of steroid hormones, vitamin D, and bile acids, and is involved in various biological processes (2). However, the imbalance of cholesterol metabolism can lead to many diseases, such as atherosclerosis, fatty liver and lung diseases (3). Cholesterol is the most abundant neutral lipid in alveolar surfactants, with innate immune function that helps control inflammation and prevent lung infections (4). Therefore, cholesterol homeostasis is crucial for maintaining the basic biological functions of the lungs, and studying the regulatory mechanisms of cholesterol metabolism in the lungs is of great significance for understanding and preventing these diseases (5, 6).

ABCA1 is a membrane protein primarily responsible for transporting cholesterol and phospholipids from the intracellular to the extracellular space, ultimately forming high-density lipoprotein (HDL), which plays a critical role in cholesterol reverse transport (7). Defects in ABCA1 can lead to intracellular cholesterol accumulation, thereby increasing the risk of cardiovascular disease (8). An increasing number of studies indicate that ABCA1 is not only associated with cardiovascular disease, but may also be involved in the occurrence and development of lung diseases (9). For example, studies have found significant changes in the expression levels of ABCA1 in lung adenocarcinoma tissues, which are closely related to cholesterol levels and metabolism (10, 11). Moreover, abnormal function of ABCA1 may also affect lung inflammation response and immune regulation, thereby increasing susceptibility to lung diseases (12). Although ABCA1 has been preliminarily studied in lung related diseases, its impact on lung cholesterol homeostasis and tissue-specific regulatory mechanisms are still unclear.

In addition, CYP27A1 is one of the key enzymes in cholesterol metabolism, mainly involved in the generation of bile acids and playing an important role in cholesterol balance *in vitro* and *in vivo*. Previous studies have found that knockout of CYP27A1 can affect the synthesis of bile acids (13). In the alternative pathway of bile acid synthesis, CYP27A1 is activated to generate 27 hydroxycholesterol (27-HC), which directly participates in lipid metabolism. It can affect the development of non-alcoholic fatty liver disease and hepatocellular carcinoma by regulating cholesterol metabolism, promoting the synthesis of bile acids (14, 15). Although lung tissue does not directly participate in lipid metabolism like the liver, it is equally important for maintaining cholesterol homeostasis. CYP27A1 catalyzed 27-HC can activate liver X receptors (LXR), promote cholesterol excretion by ABCA1 (16, 17), and thus inhibit the growth and proliferation of lung cancer. But there are also studies that have shown through *in vitro* experiments that 27-HC promotes the proliferation and migration of lung adenocarcinoma (18). However, the study of CYP27A1 and ABCA1 in lung cholesterol homeostasis still needs further clarification, which may be a key point in explaining their different manifestations in different pathological processes.

Our previous study confirmed that CYP27A1 regulates ABCA1 expression through its metabolic product 27-HC, which activates the LXR signaling pathway. Based on this mechanism, this study was to investigate the tissue specificity of CYP27A1 and ABCA1 in the regulation of cholesterol homeostasis in liver and lung tissues under HCD. Meanwhile, the application of the CYP27A1 KO mouse model allows the retention of ABCA1 expression, thereby enabling the investigation of ABCA1 expression changes in the liver and lung after CYP27A1 KO, its relationship with cholesterol metabolism, and the importance of ABCA1 in the tissue-specific regulation of pulmonary cholesterol homeostasis.

## 2 Experimental animals and methods

### 2.1 Experimental animals

*CYP27A1*^(−/−)^ mice were bred from C57BL/6J background *CYP27A1*^(+/−)^ heterozygous mice (five females, eight males, 009106 Bar Harbor, ME) purchased from Jackson Laboratories in the United States. All animals were raised in the Experimental Animal Center of Chongqing Medical University in an SPF level barrier environment, free of specific pathogens, with room temperature ranging from 18°C to 28°C, relative humidity of 40% to 70%, 12 h of light/12 h of darkness, and sufficient food and water.

### 2.2 Mouse genotype identification

Mouse genotyping was performed by digesting toe tissue using a commercial genotyping kit (Beyotime, D7283S). Polymerase chain reaction amplifier (PCR) amplification was performed using specific primer sequences, reaction components, and thermal cycling conditions as listed in Table 1. Dissolve 0.5 g of agarose in 50 mL of TAE solution, microwave heating for dissolution, add 4 μL of ethidium bromide after slightly cooling, mix well and pour into the gel making rack. The PCR product was mixed with 1 × DNA Loading Buffer, and 8 μL samples were loaded on each well. After 30 min of 120 V electrophoresis, gel imaging analysis was performed.

**Table 1:** Primer sequences, amplification system components, and reaction program for mouse genotyping.

Primer sequence	
Primer 1	AAACTCCCGGATCATCACAG
Primer 2	CTCACCCTTGACAGCATCAG
Primer 3	GCCAGAGGCCACTTGTGTAG

Amplification system	
Primer 1	1 μL
Primer 2	1 μL
Primer 3	1 μL
2× Taq Master Mix	10 μL
RNase-free H_2_O	6 μL
cDNA	1 μL

Reaction procedure	
Temperature and time	Number of cycles
94°C 2 min	
94°C 15 s	$\}\times 10$
65°C 15 s (decrease by 0.5°C per cycle)	
68°C 30 s	
94°C 15 s	$\}\times 28$
60°C 15 s	
72°C 30 s	
72°C 2 min	
10°C save	

### 2.3 Establishment of mouse model

**Figure 1:**
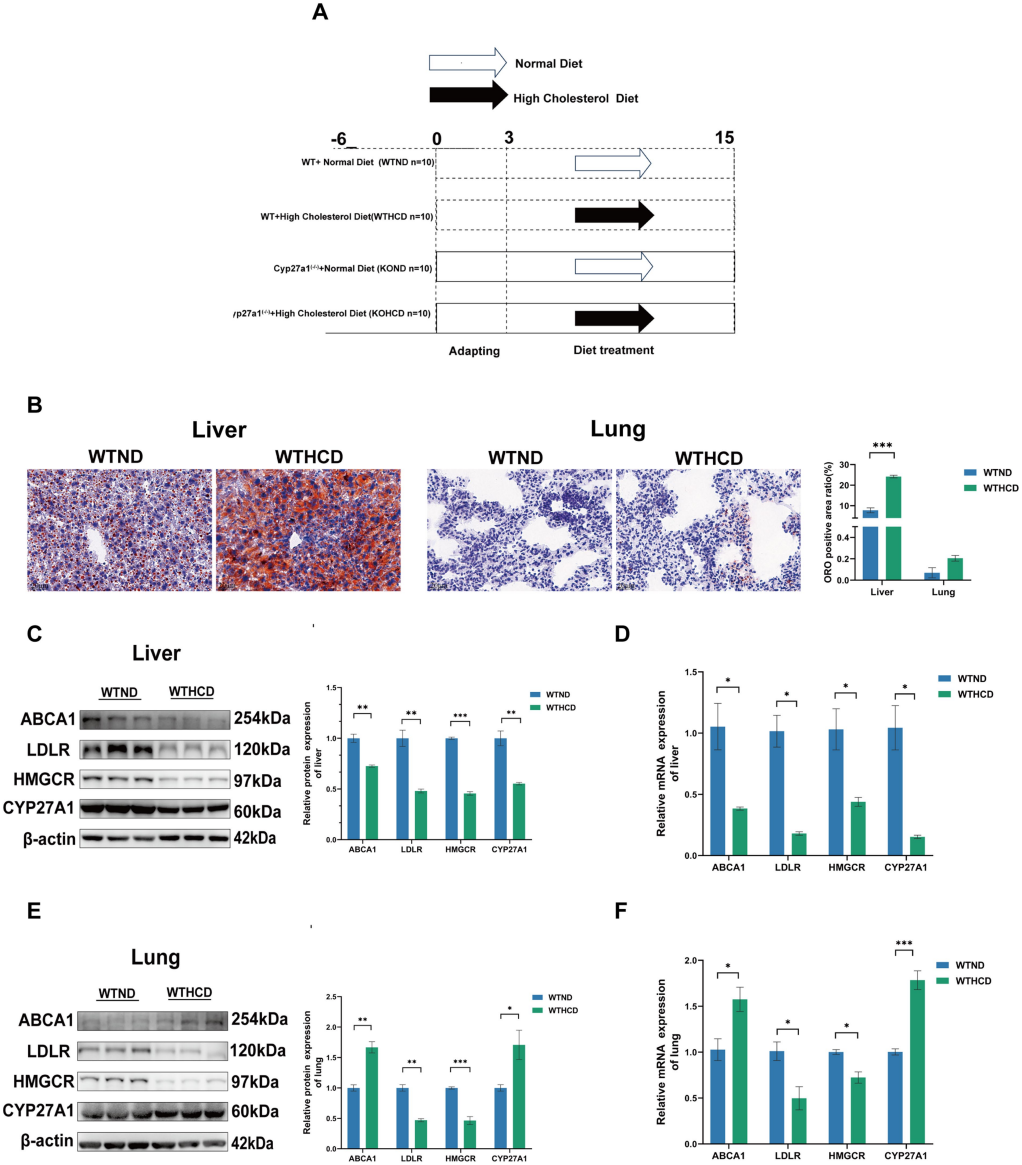
Tissue specific expression of CYP27A1 and ABCA1 under HCD. **(A)** Modeling schematic diagram of *CYP27A1*^(−/−)^ and WT mice with C57BL/6J background receiving ND and HCD for 12 weeks, respectively. **(B)** ORO staining of liver and lung tissues in WT mice fed with ND or HCD, respectively. **(C)** Protein levels of ABCA1, LDLR, HMGCR, and CYP27A1 in liver tissues. **(D)** Relative mRNA levels of ABCA1, LDLR, HMGCR, and CYP27A1 in liver tissues. **(E)** Protein levels of ABCA1, LDLR, HMGCR, and CYP27A1 in lung tissues. **(F)** Relative mRNA levels of ABCA1, LDLR, HMGCR, and CYP27A1 in lung tissues. *n* = 3–5 mice/group. Data are presented as mean ± SEM. ^*^p < 0.05, ^**^p < 0.01, and ^***^p < 0.001.

In the experiment, the normal diet (ND, cholesterol free and bile salt), was used as a standard laboratory animal diet. The high-cholesterol diet (HCD, containing 1.25% cholesterol and 4.5% bile salts) was applied to simulate metabolic disorders induced by excessive cholesterol intake. This dietary formulation has been widely used to establish disease models such as non-alcoholic fatty liver disease (NAFLD), atherosclerosis, and cholesterol metabolism disorders, owing to its experimental reproducibility and pathological relevance (19). All feed was purchased from Research Diets in Germany. See Table 2 for the list of dietary ingredients. Male CYP27A1 KO mice and WT mice were divided into four groups: WTND group (WT mice fed ND), WTHCD group (WT mice fed HCD), KOND group (KO mice fed ND), and KOHCD group (KO mice fed HCD). All mice were 6–10 weeks old, with 10 mice in each group. After 3 weeks of adaptive feeding, the mice were given diet intervention for 12 weeks, and weighed at a fixed time every week (Figure 1A).

**Table 2:** Proportion and energy content of mouse feed ingredients in ND and HCD.

Product	ND (D12102C)		HCD (D12109C)	
	gm	kcal	gm	kcal
%				
Protein	19	20	23	20
Carbohydrate	67	70	45	40
Fat	4	10	20	40
Ingredient				
Casein, lactic	200	800	200	800
L-Cystine	3	12	3	12
Corn starch	375	1,500	212	848
Maltodextrin10	125	500	71	284
Sucrose	200	800	113	452
Cellulose, BW200	50	0	50	0
Soybean oil	25	225	25	225
Cocoa butter	20	180	155	1,395
Mineral mix S10021	10	0	10	0
Dicalcium phosphate	13	0	13	0
Calcium carbonate	5.5	0	5.5	0
Potassium citrate	16.5	0	16.5	0
Vitamin mix V10001	10	40	10	40
Choline bitartrate	2	0	2	0
Cholesterol	0	0	11.25	0
Sodium cholate	0	0	4.5	0

### 2.4 Transcriptome analysis

Lung tissue samples (50 mg) were collected from mice in four experimental groups: WTND group (*n* = 5), WTHCD group (*n* = 5), KOND group (*n* = 4), and KOHCD group (*n* = 4). Total RNA was extracted using Trizol reagent, and RNA concentration and purity were measured and assessed using a NanoDrop 2000 spectrophotometer (Thermo Fisher Scientific, United States). Library construction was performed using the Illumina NovaSeq Reagent Kit, and sequencing was carried out on an Illumina NovaSeq Xplus (Illumina, United States). The transcriptomic sequencing generated 126.15 Gb of clean data, with each sample yielding more than 6.12 Gb of clean data. The Q_30_ base quality exceeded 96.1%, meeting the requirements for comprehensive gene expression analysis. All samples were submitted to Shanghai Meiji Biomedical Technology Co., Ltd. for RNA sequencing (Project No. MJ20240228062). Data analysis is conducted on the Majorbio cloud platform.^1^ The software used for gene differential expression analysis is DESeq2.^2^ The software used for KEGG pathway enrichment analysis is KOBAS.^3^

### 2.5 Serum collection and parameter analyses

Mice were fasting before sample collection. Before euthanasia, 1% Pentobarbital Sodium was injected intraperitoneally at the concentration of 0.1 mL/10 g body weight. Then, cardiac blood was collected using a 1 mL syringe. After 2–3 h of storage at room temperature, the blood was centrifuged at 400 × g for 15 min, and the serum was collected and stored in a −80°C refrigerator before use. The serum was sent to the Affiliated Hospital of Chongqing Medical University, and the blood lipid spectrum, including total cholesterol (TC), triglyceride (TG) and high/low density lipoprotein cholesterol (HDL-C and LDL-C), was determined by automatic biochemical analyzer.

### 2.6 Hematoxylin–eosin staining

Liver and lung tissues were fixed in 4% paraformaldehyde. The samples were embedded in paraffin and sectioned at a thickness of 4 μm for hematoxylin–eosin staining (HE). Sections were baked overnight at 60°C, then dewaxed in xylene and rehydrated through a graded ethanol series (100%-95%-80%-70%). After rinsing in distilled water three times, the sections were immersed in hematoxylin solution for 30 s and washed with running tap water. Nuclear staining intensity was assessed under a microscope and adjusted using 1% acid alcohol if necessary. The sections were then counterstained with eosin solution for 10 s at room temperature. Finally, the sections were dehydrated and mounted with neutral resin. Digital imaging was performed using a Pannoramic DESK digital slide scanner (3DHISTECH, Hungary).

### 2.7 Oil Red O stain

Frozen liver and lung tissue sections stored at −20°C were rewarmed for 10 min. The sections were fixed in 10% formalin for 20 min, then rinsed on a shaker in distilled water. Afterward, the sections were differentiated in 60% isopropanol for 1 min and stained with freshly prepared Oil Red O stain (ORO) working solution at 37°C in the dark for 30 min. Following staining, the sections were immersed in 60% isopropanol for 1–2 min, and differentiation was assessed microscopically. After differentiation, the sections were washed in distilled water for 10 min. Hematoxylin counterstaining was performed for 20 s, followed by thorough rinsing with running water. Finally, the sections were sealed with glycerol gelatin.

### 2.8 Immunohistochemistry (IHC)

Paraffin-embedded tissue sections were baked at 60°C for 2–4 h, dewaxed in xylene I and II for 15 min each, and rehydrated through a graded ethanol series (100%-95%-80%-70%) for 5 min at each step. Antigen retrieval was performed in citrate buffer. Immunohistochemical staining was conducted according to the instructions of the commercial kit (Fuzhou Maixin, KIT-9710). Diaminobenzidine (DAB) was used for color development, followed by hematoxylin counterstaining for 20 s. The sections were dehydrated through graded ethanol (70%-80%- 95%-100%), cleared in xylene I and II for 30 min each, and mounted with neutral resin.

### 2.9 Quantitative real-time PCR

Total RNA was extracted from tissue cells using Trizol reagent (DP424, Tiangen), followed by reverse transcription using 1,000 ng of total RNA (RR047A, Takara). The system was divided into a gDNA removal system and a reverse transcription system, with both systems having a volume of 10 μL. Perform quantitative real-time PCR (qRT-PCR) using SYBR Green qPCR premix (HY-K053, MCE). Calculate the relative mRNA expression fold changes based on endogenous β-actin. The specific primer sequences are shown in Table 3.

**Table 3:** List of primers for mouse qRT-PCR reactions.

Gene	Forward (5′–3′)
*Cyp27a1*-forward (5′–3′)	TGGTTCCCACAAACTCCCGGATCAT
*Cyp27a1*-reverse (5′–3′)	CCATAGCCAAAGGGCACAGAGCCAA
*Hmgcr*-forward (5′–3′)	AGCTTGCCCGAATTGTATGTG
*Hmgcr*-reverse (5′–3′)	TCTGTTGTGAACCATGTGACTTC
*Ldlr*-forward (5′–3′)	TGACTCAGACGAACAAGGCTG
*Ldlr*-reverse (5′–3′)	ATCTAGGCAATCTCGGTCTCC
*Abca1*-forward (5′–3′)	CGTTTCCGGGAAGTGTCCTA
*Abca1*-reverse (5′–3′)	GCTAGAGATGACAAGGAGGATGGA
*Tnf-α*-forward (5′–3′)	AGGCACTCCCCCAAAAGATG
*Tnf-α*-reverse (5′–3′)	GCCATTTGGGAACTTCTCAT
*Il-1β*-forward (5′–3′)	TCGCAGCAGCACATCAACAAGA
*Il-1β*-reverse (5′–3′)	AGGTCCACGGGAAAGACACAGG
*Il-10*-forward (5′–3′)	AGCCTTATCGGAAATGATCCAGT
*Il-10*-reverse (5′–3′)	GGCCTTGTAGACACCTTGGT
*β-actin*-forward (5′–3′)	TGAGCTGCGTTTTACACCCT
*β-actin*-reverse (5′–3′)	GCCTTCACCGTTCCAGTTTT

### 2.10 Western blot

Total protein was extracted from cells or tissues using a lysis buffer containing protease inhibitors, phosphatase inhibitors, and PMSF. Protein concentration was determined using the bicinchoninic acid (BCA) method. Equal amounts of protein were separated by SDS-PAGE and transferred to PVDF membranes. The membranes were blocked with 5% non-fat milk at 37°C for 1 h, then incubated overnight at 4°C with primary antibodies. After washing, the membranes were incubated with secondary antibodies at 37°C for 1 h. Signals were detected using ECL substrate (BG0015, Bioground Biotech Co., Ltd.) and visualized with a chemiluminescence imaging system. The primary antibodies used in this study included: ABCA1 (1:1,000, Abclonal), LDLR (1:1,000, Abclonal), HMGCR (1:1,000, Abclonal), CYP27A1 (1:1,000, Abcam), sterol regulatory element-binding Protein 2 (SREBP2) (1:1,000, Proteintech), ATP-binding cassette subfamily G1 (ABCG1), (1:1,000, Proteintech), p-NF-κB/NF-κB (1:1,000, CST), ACTIN (1:1,000, Proteintech).

### 2.11 Immunofluorescence staining

Paraffin-embedded tissue sections were baked at 60°C for 2–4 h, then dewaxed in xylene I and II for 20 min each. The sections were rehydrated through a graded ethanol series (100%-95%-80%-70%) for 5 min at each step, followed by antigen retrieval in citrate buffer for 30 min. Multiplex fluorescence staining was performed according to the manufacturer’s instructions of the staining kit (AFIHC024, AiFang Biotech). Finally, DAPI was applied in the dark for 5 min, and the sections were sealed with an anti-fade mounting medium CD68 (1:100, boster), SFTPC (1:100, Proteintech), ABCA1(1:100, Proteintech).

### 2.12 Statistical analysis

This study used SPSS 25.0 for data analysis, GraphPad Prism 8.0 for plotting, Student’s *t*-test or Wilcoxon rank sum test for comparison between two groups, and one-way ANOVA or Kruskal–Wallis *H* test for comparison between three or more groups. All statistical analyses were two-tailed with a significance level set at 0.05. Data were presented as mean ± SEM. Differences were considered statistically significant at *p* < 0.05. Statistical significance was indicated as follows: ^*^p < 0.05, ^**^p < 0.01, and ^***^p < 0.001.

## 3 Results

### 3.1 Tissue-specific expression of CYP27A1 and ABCA1 under HCD

ORO staining revealed distinct cholesterol metabolic responses in the liver and lung tissues of WT mice fed HCD.

In the liver, compared with the ND group, the HCD group exhibited markedly enlarged lipid droplets and significant lipid accumulation (Figure 1B). In contrast, no obvious lipid droplets were observed in lung tissue, and the difference was not statistically significant. Western blot (WB) analysis further showed that in the liver, HCD feeding led to a feedback downregulation of the low-density lipoprotein receptor (LDLR) and 3-hydroxy-3-methylglutaryl-CoA reductase (HMGCR), key regulators of cholesterol uptake and synthesis. Concurrently, the expression of CYP27A1 and ABCA1, both involved in cholesterol clearance, was also significantly downregulated (Figure 1C).

In contrast, the lung tissue exhibited a distinct pattern. Similar to the liver, LDLR and HMGCR were downregulated; however, the expression of CYP27A1 and ABCA1 was significantly upregulated in the HCD group (Figure 1E). This tissue-specific pattern was further confirmed by qRT-PCR (Figures 1D,F): CYP27A1 and ABCA1 mRNA levels were downregulated in the liver, while significantly upregulated in the lung under HCD. These findings suggest that CYP27A1 and ABCA1 may participate in tissue-specific regulation of cholesterol metabolism in WT mice. Given that CYP27A1 acts upstream of ABCA1, we next employed whole-body knockout of CYP27A1 to further investigate its role in tissue-specific metabolic regulation.

### 3.2 Effects of HCD and CYP27A1 KO on body weight, organ ratio, and blood lipids in C57BL/6J mice

**Figure 2:**
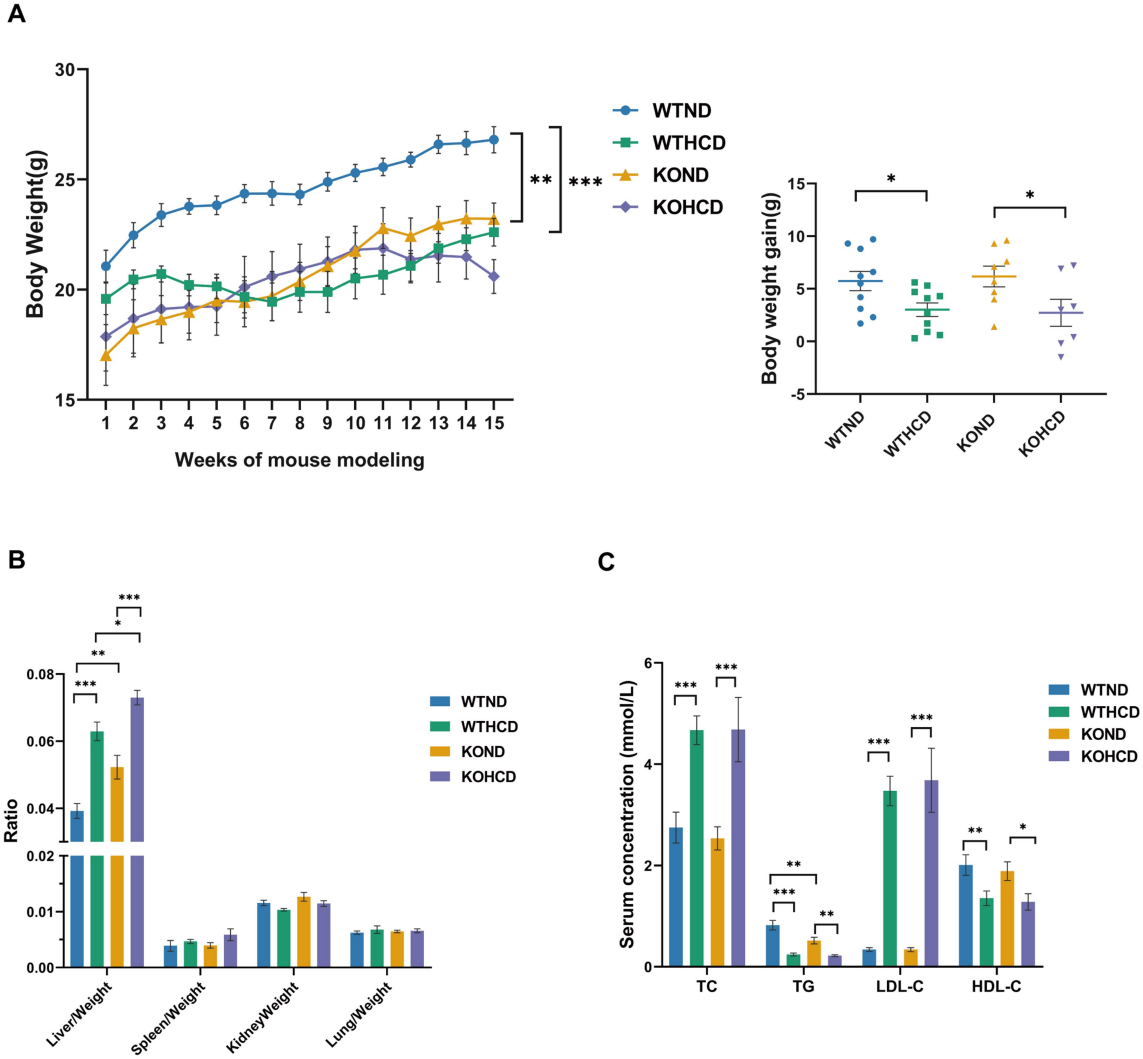
Effects of HCD and CYP27A1 KO on body weight, organ ratio, and blood lipids in C57BL/6J mice. **(A)** Body weight growth curves of mice fed with ND and HCD. And body weights of mice measured once a week. **(B)** Organ ratio of mice in four groups. **(C)** Levels of total TC, TG, LDL, and HDL in serum. *n* = 7–10 mice/group. Data are presented as mean ± SEM. ^*^p < 0.05, ^**^p < 0.01, and ^***^p < 0.001.

Body weight was monitored weekly throughout the 12 weeks feeding period. At the end of the experiment, major organs including the liver, lung, kidney, and spleen were collected and weighed. Compared with the WTND group, mice in the WTHCD group exhibited a significant reduction in body weight (Figure 2A), while liver body weight ratio were significantly increased. In contrast, no significant changes were observed in the weights of other organs such as the lung or kidney (Figure 2B). Serum lipid analysis revealed that HCD feeding elevated TC and LDL-C levels, while TG and HDL-C levels in WT mice (Figure 2C).

In CYP27A1 KO mice, compared with the KOND group, the KOHCD group resulted in an increased liver body weight ratio with lipid level changes similar to those observed in WT mice.

Under ND conditions, KO mice exhibited lower body weight than WT mice, along with an increased liver body weight ratio. No significant differences were observed in the weights of other organs such as the lung and kidney. Serum TG levels were markedly reduced in KO mice, while other lipid parameters remained unchanged.

In conclusion, HCD had a significant impact on body weight, liver index, and lipid profiles in both WT and KO mice. However, the global metabolic phenotype of CYP27A1 KO was relatively mild, it may still influence tissue-specific lipid regulation under both physiological and dietary stress conditions.

### 3.3 Specific effect of HCD and CYP27A1 KO on lipid accumulation in liver and lung tissues

**Figure 3:**
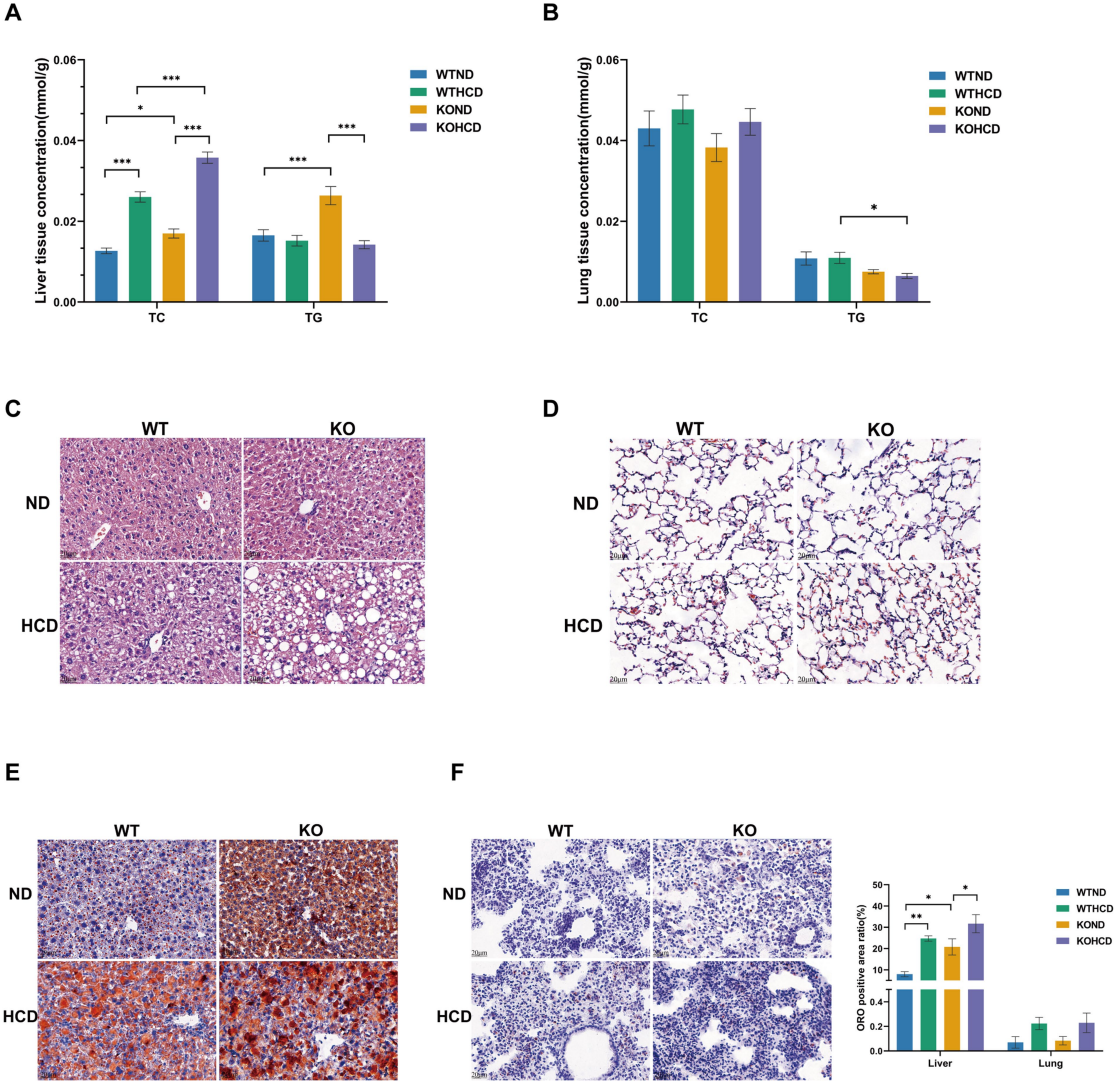
Specific effect of HCD and CYP27A1 KO on lipid accumulation in liver and lung tissues. **(A)** Levels of total TC, TG in liver tissues. **(B)** Levels of total TC, TG in lung tissues. **(C)** HE staining of liver tissues of *CYP27A1*^(−/−)^ and WT mice receiving ND and HCD, respectively. **(D)** HE staining of lung tissues of *CYP27A1*^(−/−)^ and WT mice receiving ND and HCD, respectively. **(E)** ORO staining of liver tissue in *CYP27A1*^(−/−)^ and WT mice receiving ND and HCD, respectively. **(F)** ORO staining of lung tissue in *CYP27A1*^(−/−)^ and WT mice receiving ND and HCD, respectively. Scale bars = 20 μm. *n* = 3–5mice/group. Data are presented as mean ± SEM. ^*^p < 0.05, ^**^p < 0.01, and ^***^p < 0.001.

After 12 weeks of HCD feeding, measurements of TC and TG in liver and lung tissues were performed (Figure 3A). compared with the ND group, HCD significantly increased hepatic TC levels in WT mice. Consistently, HE staining revealed increased lipid droplets in the liver (Figure 3C), and ORO staining showed abundant lipid droplets (Figure 3E), indicating notable hepatic lipid accumulation. In contrast, the lung tissue exhibited metabolic tolerance to HCD (Figures 3B,D,F), although TC levels in the lung showed an increasing trend, the difference was not statistically significant, and ORO staining revealed no obvious lipid accumulation.

In CYP27A1 KO mice, compared with the KOND group, the KOHCD group further increased the TC level in liver tissue, while the TG level decreased. HE staining revealed a marked increase in hepatic lipid droplets, and ORO staining confirmed extensive lipid droplet accumulation, indicating exacerbated hepatic steatosis. However, the lung tissues of KO mice showed no significant lipid accumulation, either in TC levels or ORO staining, suggesting metabolic tolerance to both HCD and CYP27A1 deficiency.

Under ND conditions, KO mice displayed elevated hepatic TC and TG levels compared with WT mice. Mild lipid droplet was observed in the liver, and ORO staining revealed an increased number of lipid droplets, indicating enhanced hepatic lipid accumulation. In contrast, TC levels and ORO staining in the lungs of KO mice did not show evidence of lipid accumulation.

Taken together, both HCD and CYP27A1 deficiency promoted lipid accumulation in the liver, while the lung tissue exhibited metabolic tolerance, with no significant lipid deposition.

### 3.4 Tissue specificity of cholesterol metabolism and ABCA1 expression in liver and lung tissues

**Figure 4:**
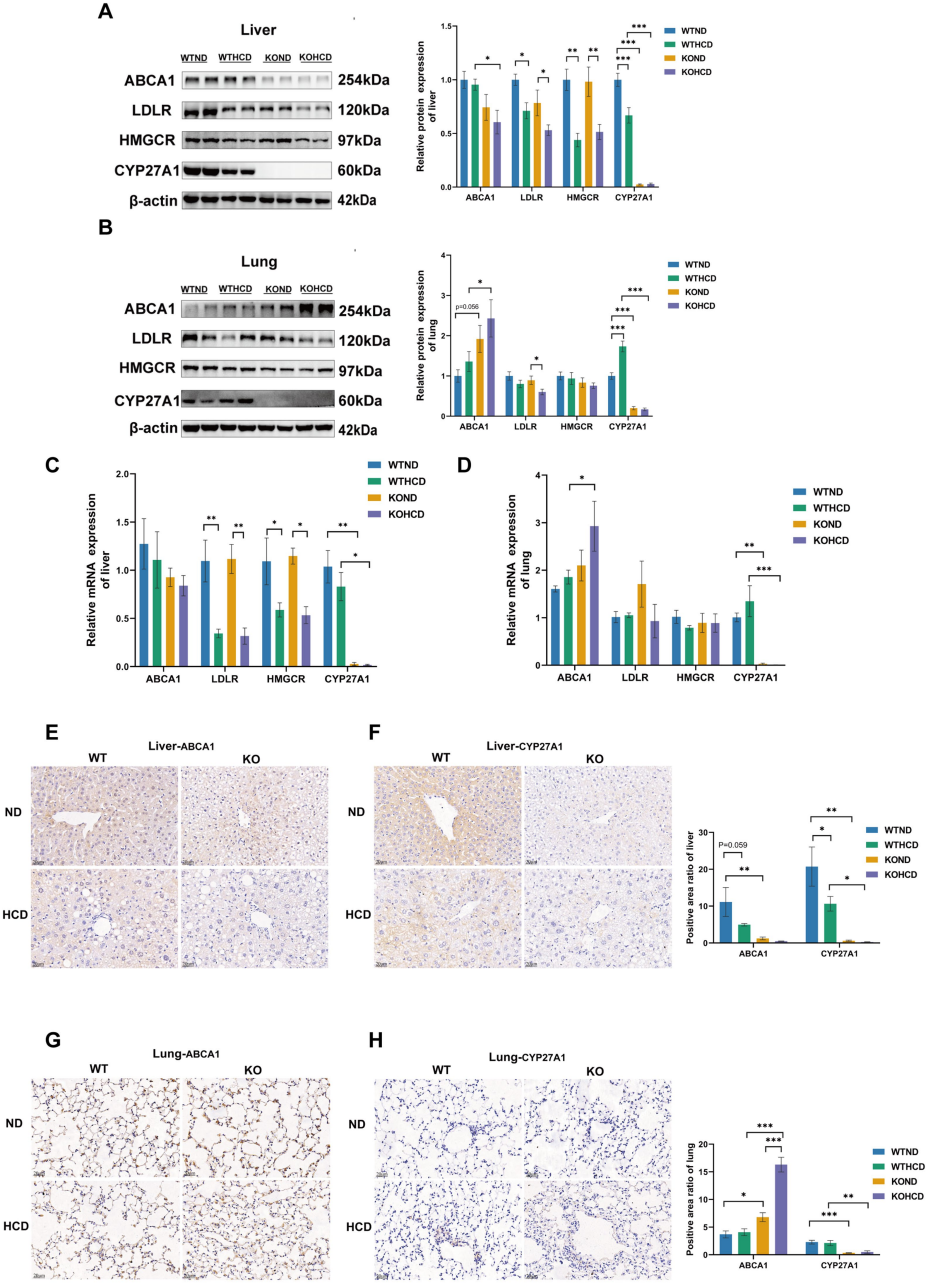
Differential expression of cholesterol metabolism-related proteins and ABCA1 in liver and lung tissues. **(A)** Protein levels of ABCA1, LDLR, HMGCR, and CYP27A1 in liver tissues. **(B)** Protein levels of ABCA1, LDLR, HMGCR, and CYP27A1 in lung tissues. **(C)** Relative mRNA levels of ABCA1, LDLR, HMGCR, and CYP27A1 in liver tissues. **(D)** Relative mRNA levels of ABCA1, LDLR, HMGCR, and CYP27A1 in lung tissues. **(E)** Immunohistochemical staining of ABCA1 in liver tissue with *CYP27A1*^(−/−)^ and WT mice receiving ND and HCD, respectively. **(F)** Immunohistochemical staining of CYP27A1 in liver tissue with *CYP27A1*^(−/−)^ and WT mice receiving ND and HCD, respectively. **(G)** Immunohistochemical staining of ABCA1 in lung tissue with *CYP27A1*^(−/−)^ and WT mice receiving ND and HCD, respectively. **(H)** Immunohistochemical staining of CYP27A1 in lung tissue with *CYP27A1*^(−/−)^ and WT mice receiving ND and HCD, respectively. Scale bars = 20 μm. *n* = 3–5 mice/group. Data are presented as mean ± SEM. ^*^p < 0.05, ^**^p < 0.01, and ^***^p < 0.001.

Although pronounced lipid accumulation was observed in the liver under either CYP27A1 KO or HCD conditions, no similar changes were found in the lung tissue (Figures 3C–F). To investigate the underlying cause, we examined the expression of genes and proteins involved in cholesterol uptake, synthesis, and efflux. WB and qRT-PCR analyses revealed that ABCA1 expression exhibited a tissue-specific pattern (Figures 4A–D). Under both ND and HCD conditions, CYP27A1 KO downregulated ABCA1 expression in the liver, whereas ABCA1 expression in the lung was significantly upregulated. In addition, no significant differences were observed in the expression levels of ABCG1 and SREBP2 in either the liver or lung tissues among the four groups (Supplementary Figures 1A,B). Meanwhile, in the liver, compared with the KOND group, the expression levels of LDLR and HMGCR protein and mRNA in the KOHCD group were significantly decreased. In lung tissue, compared with the KOND group, the expression of LDLR in the KOHCD group was significantly decreased, while there were no significant differences in other groups. In addition, no significant differences in LDLR or HMGCR protein levels were found in either liver or lung tissues after CYP27A1 KO. Immunohistochemical results further supported the tissue specific regulation of ABCA1 (Figures 4E–H), showing a progressive decrease in the liver and a gradual increase in the lung.

**Figure 5:**
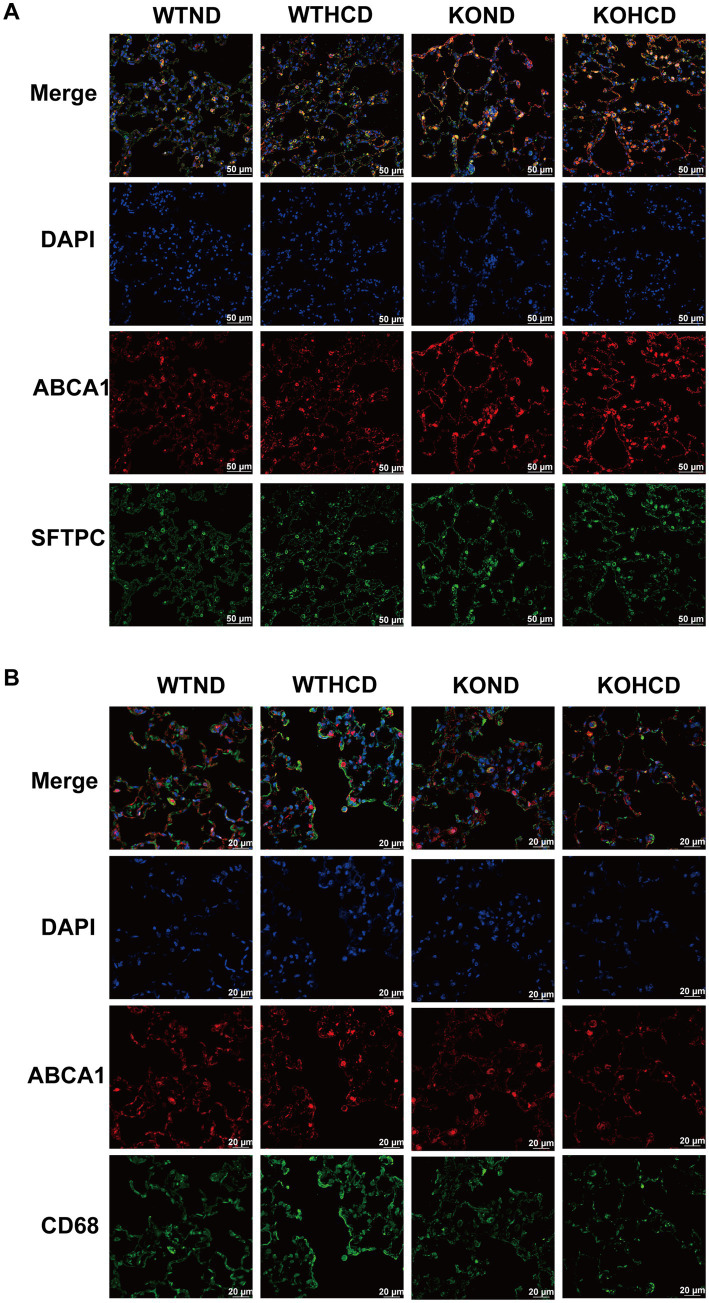
Subcellular localization of ABCA1 in lung tissue. **(A)** Co-staining of ABCA1 (red) and SFTPC (green, alveolar type II marker); nuclei stained with DAPI (blue). **(B)** Co-staining of ABCA1 (red) and CD68 (green, macrophage marker) to determine cell-type-specific expression. *n* = 3–5 mice/group.

Immunofluorescence analysis (Figures 5A,B) showed that ABCA1 was abundantly expressed in lung tissue and primarily localized to alveolar type II epithelial cells. In contrast, ABCA1 expression in macrophages did not show a clear increase. These findings suggest that alveolar type II epithelial cells may play a critical role in pulmonary cholesterol homeostasis, potentially by promoting cholesterol efflux to prevent excessive lipid accumulation in the lung.

In summary, ABCA1 expression in the liver and lung exhibited tissue specific differences under HCD or CYP27A1 KO conditions, which may be related to distinct physiological functions and regulatory mechanisms of ABCA1 in different cell types.

### 3.5 Preliminary exploration of the mechanism of ABCA1 upregulation in lung tissue

**Figure 6:**
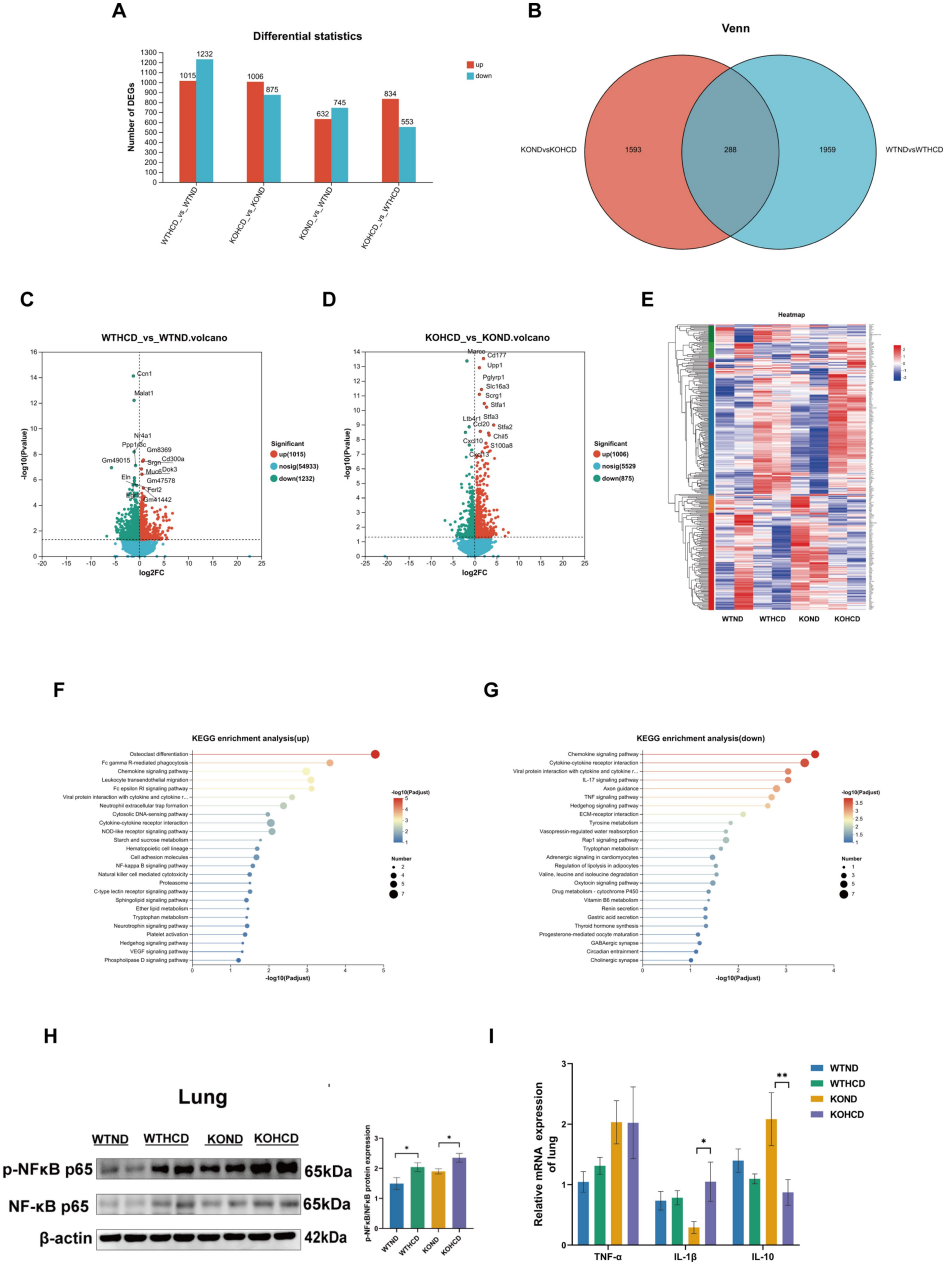
Preliminary exploration of the mechanism of ABCA1 upregulation in lung tissue. **(A)** The number of differentially expressed genes compared between two groups. **(B,E)** The differential genes between WTND vs. WTHCD, KOND vs. KOHCD were analyzed by Venn analysis, resulting in 288 common differential genes and gene heatmaps genes. **(C,D)** Volcanic diagram of differentially expressed genes in the lungs of WTND vs. WTHCD, KOND vs. KOHCD mice. The color represents *p* < 0.05 and the fold change >1 (red), *p* < 0.05, the fold change is <1 (green) and not significant (blue). **(F,G)** The top 25 signaling pathways in KEGG enrichment analysis. **(H)** Protein levels of p-NFκB, NFκB in lung tissues. **(I)** Relative mRNA levels of TNF-α, IL-1β, and IL-10 in lung tissues. *n* = 3–5 mice/group. Data are presented as mean ± SEM. ^*^p < 0.05 and ^**^p < 0.01.

To explore the molecular mechanism of ABCA1 upregulation in lung tissue and its potential role in cholesterol clearance, RNA-seq analysis was conducted on lung tissues from WT and CYP27A1 KO mice fed with either ND or HCD. Differentially expressed genes were screened using the criteria of |log_2_FC| >0 and *p*-value <0.05. The results showed distinct gene expression differences in lung tissues under different dietary conditions (Figure 6A). In the volcano plots, compared with the WTND group, the WTHCD group showed 1,015 upregulated genes and 1,232 downregulated genes. Compared with the KOND group, the KOHCD group showed 1,006 genes upregulated and 875 genes downregulated (Figures 6C,D). Venn diagram analysis between the DEGs of WTND vs. WTHCD and KOND vs. KOHCD revealed 288 Common genes (Figure 6B). Further clustering and KEGG enrichment analysis (Figure 6E) showed that the upregulated genes were significantly enriched in pathways associated with immune regulation, such as Fc gamma receptor (FcγR)-mediated phagocytosis, the NF-κB signaling pathway, and other signal transduction processes. In contrast, the downregulated genes were enriched in pathways related to immune signaling modulation, metabolic regulation, endocrine functions, and cancer-related biological processes (Figures 6F,G).

### 3.6 Expression of NF-κB signaling pathway in mice under different treatments

NF-κB, as a classical inflammation pathway, has been reported to regulate ABCA1 expression (20). Therefore, we selected the NF-κB pathway for subsequent validation. We examined the expression of the NF-κB signaling pathway and its related inflammatory genes using WB and qRT-PCR (Figures 6H,I). The results were consistent with the RNA-seq data: both HCD and CYP27A1 KO enhanced the expression of NF-κB pathway proteins, which paralleled the expression trend of ABCA1 observed (Figure 4B). Meanwhile, proinflammatory cytokines such as tumor necrosis factor-α (TNF-α) and interleukin-1β (IL-1β) showed an increasing trend in lung tissue following HCD treatment, while the expression level of the anti-inflammatory cytokine interleukin-10 (IL-10) was decreased.

## 4 Discussion

This study used the liver as a comparative reference to systematically analyze the effects of HCD and CYP27A1 KO on pulmonary cholesterol metabolism in mice, and further explore the potential regulatory mechanisms of ABCA1 in maintaining cholesterol homeostasis in the lung.

Our study found that in WT mice, compared with ND group, HCD can promote the accumulation of cholesterol and other lipids in the liver, which is consistent with previous research results (21–23). However, weight loss was observed in the HCD group. This may be related to metabolic stress induced by the HCD, which could reduce food intake or alter feeding behavior. Additionally, high cholesterol intake could impair liver function, affecting lipid digestion and absorption, leading to weight loss (24). These potential mechanisms require further investigation. In this study, TG levels in the liver decreased under HCD, while lipid droplet accumulation increased, as shown by HE and ORO staining. This may reflect abnormal lipid redistribution or retention. Under metabolic stress, hepatocytes may convert TG into other lipid forms or enhance lipid storage to reduce lipotoxicity (25, 26).

After 12 weeks of HCD feeding, although hepatic cholesterol synthesis and uptake decreased, efflux was also impaired, ultimately leading to cholesterol buildup in the liver. This effect was further aggravated by CYP27A1 KO, highlighting the important role of CYP27A1 in maintaining hepatic cholesterol balance (13, 27, 28). Similar findings have been reported in other studies, where mice with reduced CYP27A1 expression developed hepatomegaly, lipid accumulation, and metabolic abnormalities (29, 30). Moreover, our data showed that regardless of dietary condition, ABCA1 protein expression in the liver was downregulated in CYP27A1 KO mice compared with WT controls. As a downstream efflux transporter, reduced ABCA1 expression likely impaired cholesterol elimination, creating a vicious cycle of metabolic dysregulation. This supports the established role of ABCA1 in hepatic lipid metabolism (30–33). Some previous studies have suggested that CYP27A1 deficiency or overexpression may influence cholesterol synthesis by upregulating LDLR and HMGCR activity (34, 35). However, we did not observe significant changes in LDLR or HMGCR expression in our study. This discrepancy may be due to the inhibitory effects of cholesterol accumulation under HCD conditions, which suppress feedback regulation of LDLR and HMGCR expression. These variations may also result from differences in experimental models, metabolic environments, or the regulatory dynamics of cholesterol homeostasis. Further studies are warranted to elucidate these mechanisms.

In addition, previous studies have demonstrated that CYP27A1 overexpression in other cell types enhances cholesterol efflux, primarily through the regulation of efflux pathways such as ABCA1 and scavenger receptor class B type I (SR-BI) (36–38).

In our study, we found that both CYP27A1 and ABCA1 expression levels were reduced in the liver under HCD feeding or CYP27A1 knockout conditions. This may be attributed to decreased production of 27-HC, a key endogenous ligand of LXR, due to CYP27A1 deficiency. As a result, LXR activation is weakened, leading to reduced ABCA1 expression, impaired cholesterol efflux capacity, and hepatic lipid accumulation and metabolic disturbances. These findings further support the critical role of CYP27A1 in maintaining hepatic cholesterol homeostasis via the LXR/ABCA1 signaling pathway (39, 40).

In contrast, the lung tissue exhibited metabolic tolerance. We observed that in WT mice, no apparent lipid accumulation occurred in the lung under HCD feeding, and cholesterol homeostasis was maintained. This may be closely related to the downregulation of LDLR and HMGCR, along with the upregulation of CYP27A1 and ABCA1 expression (16). Interestingly, even in the absence of CYP27A1, ABCA1 expression in the lungs of KO mice remained elevated. This suggests that ABCA1 may contribute to preventing excessive lipid accumulation and preserving cholesterol balance through its regulatory function. Previous studies have also shown that ABCA1 plays a central role in maintaining cholesterol homeostasis (7, 41), which aligns with our findings. Therefore, under both HCD and CYP27A1 KO conditions, we did not observe significant lipid accumulation in lung tissue. ABCA1 expression was consistently upregulated under both conditions, indicating that in the context of CYP27A1 deficiency, ABCA1 may be regulated by alternative signaling pathways. This compensatory mechanism likely ensures that cholesterol efflux remains functionally active in the lung.

The upregulation of ABCA1 in lung tissue may be influenced by other metabolic regulatory pathways. Among these, LXR is a key transcriptional factor involved in cholesterol efflux (42, 43). Under normal conditions, CYP27A1 synthesizes 27-HC, which activates LXR and subsequently upregulates ABCA1 expression to facilitate cholesterol export (18, 36). Thus, the loss of CYP27A1 is generally expected to reduce ABCA1 expression, a trend that was confirmed in liver tissue. However, in lung tissue, ABCA1 expression was paradoxically elevated in CYP27A1 KO mice fed the HCD. This finding suggests that ABCA1 expression in the lung may not be fully dependent on the LXR pathway and could be maintained by tissue-specific compensatory regulatory mechanisms.

In our preliminary exploration of the regulatory mechanisms of ABCA1 expression, we found that its upregulation may be closely associated with the activation of the NF-κB signaling pathway. Moreover, the NF-κB pathway may act in concert with other inflammatory and immune regulatory mechanisms. Previous studies have also indicated that ABCA1 expression may be linked to NF-κB activation and the and JAK/STAT signaling pathway (20, 44, 45). This mechanism of regulating ABCA1 expression through inflammation and immune signals not only enriches the understanding of the metabolic adaptability of lung tissue, but also provides a new research direction for the treatment of metabolic diseases. Moreover, emerging evidence on PI3K-Akt and sphingolipid pathways may offer complementary insight into tissue-specific metabolic regulation (46, 47).

This study has several limitations. First, we did not assess phospholipid metabolism, which is also regulated by ABCA1. Second, the regulatory mechanisms of ABCA1 remain incompletely understood and may involve complex inflammatory and immune pathways. Third, cell-based validation is lacking and should be addressed in future work. Finally, this study focused only on liver and lung tissues, and other cholesterol-metabolizing organs warrant further investigation.

## 5 Conclusion

This study revealed the tissue-specific regulatory role of ABCA1 in cholesterol metabolism in the liver and lung under conditions of HCD feeding or CYP27A1 deficiency. HCD led to the downregulation of CYP27A1 and ABCA1 expression in the liver, resulting in lipid accumulation. In contrast, increased expression of CYP27A1 and ABCA1 in the lung contributed to the maintenance of cholesterol homeostasis. CYP27A1 KO further aggravated hepatic metabolic disturbances, whereas the lung exhibited a compensatory metabolic adaptation. This adaptive response may be closely associated with increased ABCA1 expression and activation of the NF-κB signaling pathway. Overall, this study provides new evidence for the important role of ABCA1 in cholesterol metabolism and metabolic diseases, suggesting that ABCA1 may have potential clinical development value.

## Data Availability

The original contributions presented in the study are included in the article/Supplementary material, further inquiries can be directed to the corresponding author.

## Footnotes

^1^ www.majorbio.com

^2^ http://bioconductor.org/packages/stats/bioc/DESeq2/

^3^ http://kobas.cbi.pku.edu.cn/home.do

## Supplementary material

SUPPLEMENTARY FIGURE 1 Protein expression of SREBP2 and ABCG1 in liver and lung tissues. **(A)** Protein levels of SREBP2, ABCG1 in liver tissues. **(B)** Protein levels of SREBP2, ABCG1 in lung tissues.
