# Supplementary figures and images for: Tissue specific role of ABCA1 in lung cholesterol homeostasis under high-cholesterol diet
Source: Front Nutr. 2025 Jul 30;12:1649407. doi: 10.3389/fnut.2025.1649407 (PMC12343695; doi:10.3389/fnut.2025.1649407)

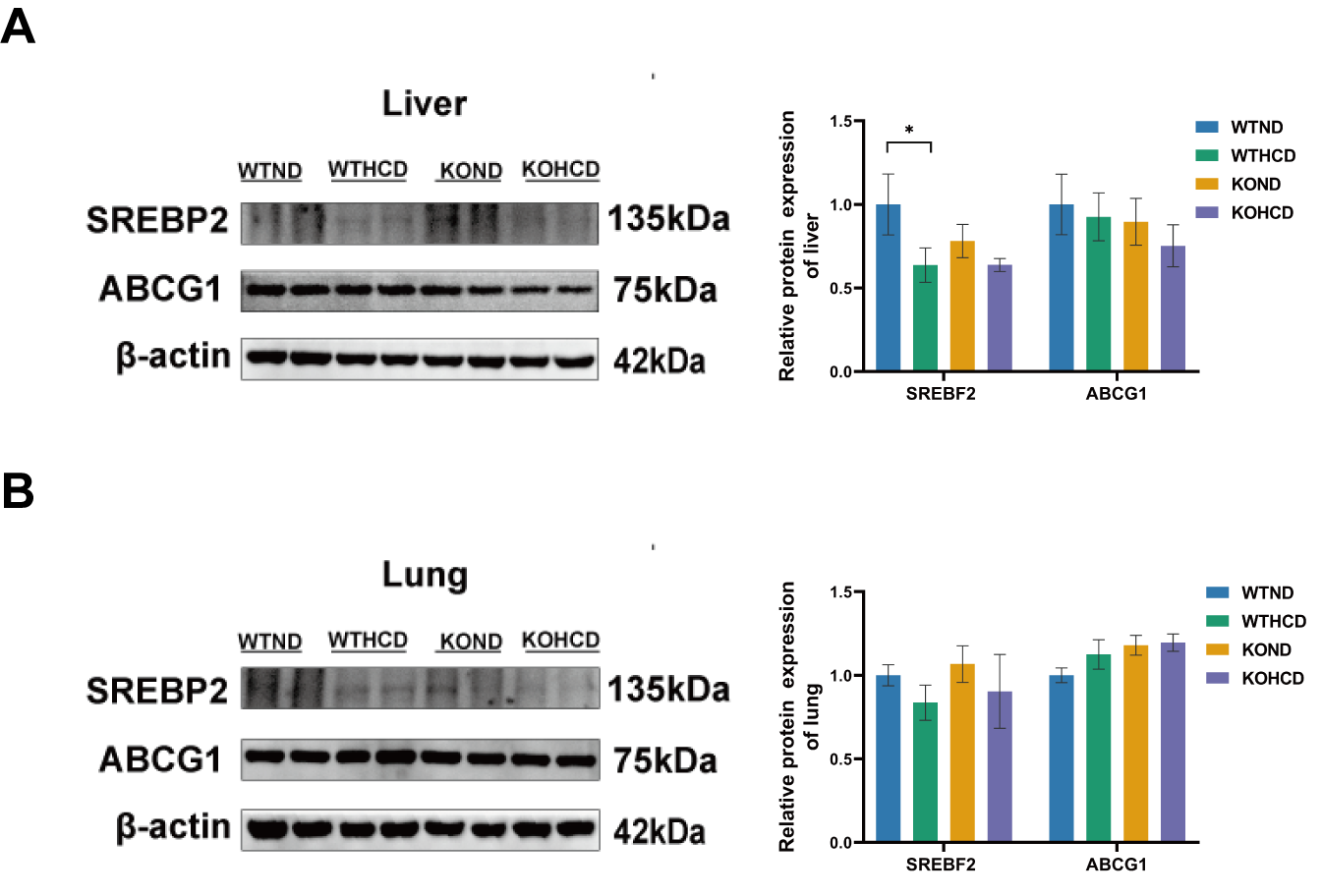

Supplement: SUPPLEMENTARY FIGURE 1 — Protein expression of SREBP2 and ABCG1 in liver and lung tissues. (A) Protein levels of SREBP2, ABCG1 in liver tissues. (B) Protein levels of SREBP2, ABCG1 in lung tissues. [file Image_1.tif]
